# Early versus delayed postoperative oral hydration in children following general anesthesia: a prospective randomized trial

**DOI:** 10.1186/s12871-020-01086-8

**Published:** 2020-07-18

**Authors:** Xiaorong Yin, Xiaoqi Zeng, Ting Wang, Binbin Dong, Menghang Wu, Anna Jia, Ling Ye

**Affiliations:** 1 Department of Anesthesiology, West China Hospital, Sichuan University/West China School of Nursing, Sichuan University, Chengdu, Sichuan Province 610041 People’s Republic of China; 2 Department of Pain Management, West China Hospital, Sichuan University, Chengdu, Sichuan Province 610041 People’s Republic of China

**Keywords:** General anesthesia, Oral intake, Postoperative nausea, Vomitting

## Abstract

**Background:**

Oral hydration has typically not been administered for between 4 and 6 h postoperative for children’s safety in China. But children are more likely to suffer from apnea, crying and agitation, wound bleeding, and other complications during the post-anesthesia recovery period because of thirsty and fear. This Prospective, randomized study sought to assess the compare the early and late oral hydration (EOH and DOH, respectively) in children following general anesthesia, with the goal of assessing relative safety and tolerability and thereby improving patient comfort.

**Methods:**

A total of 2000 children corresponding to the American Society of Anesthesiology (ASA) I-III were randomized into an EOH group (*n* = 1000) and a DOH group (n = 1000). For the former group, children were administered a small amount of drinking water following recovery of the swallowing reflex, and children’s vital signs were monitored for 20 min in a postanesthesia care unit (PACU). DOH group patients received water at 4 h following general anesthesia). All patients underwent monitoring to assess their thirst, satisfaction, oropharyngeal discomfort, nausea, and vomiting.

**Results:**

Complete data were collected from a total of 1770 patients (EOH = 832, DOH = 938) and was compared via chi-squared and t-tests as appropriate. There was no hypoxemia in either group, nor did the incidence of nausea and vomiting differ between the two groups (*P* > 0.05). The thirst score of the EOH group was significantly decreased relative to the DOH group in the children over 5 years old after drinking for 10 to 20 min (*P* < 0.05).

**Conclusions:**

For children undergoing general anesthesia, a small amount of drinking water in the early stages of recovery will not increase the incidence of nausea, vomiting, or hypoxemia, but will decrease thirst and improve satisfaction. It is important, however, that medical staff carefully monitor the swallowing reflex and vital signs of all children.

**Trial registration:**

This study was registered on the Chinese Clinical Trial Registry (ChiCTR-IOR-16008197) (http://www.chictr.org.cn/index.aspx. On April 2, 2016 the first patients was enrolled and on March 31, 2016 the trial was registered).

## Background

Compared with adult patients, children are more likely to suffer from apnea, crying and agitation, wound bleeding, and other complications during the post-anesthesia recovery period because of their own physiological and psychological characteristics [1–3]. Crying and agitation during the recovery period may cause airway spasms, severe hypoxemia leading to respiratory depression or apnea, postoperative bleeding, accidental falls, and other adverse events. It therefore represents a safety concern during anesthesia recovery period, and remains a difficult problem for nursing care [4]. Through several years of clinical observation, we found that hunger and thirst were the primary causes of agitation in children.

In order to ensure postoperative safety and prevent coughing, vomiting, and aspiration caused by drinking water following general anesthesia for non-gastrointestinal operations, postoperative oral hydration has typically not been administered for between 4 and 6 h in China [2, 3]. However, many researchers have found more rapid administration of oral hydration to be beneficial and safe [5–10]. The optimal timing for such administration of fluids, however, has remained unclear in China. For example, certain researchers have suggested that in children undergoing relatively minor operations water could safely be provided 1 h after anesthesia [8, 9]. This study sought to compare early and delayed oral hydration (EOH and DOH, respectively) after general anesthesia in order to assess the relative safety of these approaches, and to gauge their ability to decrease thirst and improve satisfaction and high-quality nursing service. Thirst, oropharyngeal discomfort, satisfaction, and incidence of nausea/vomiting, as well as the time from PACU arrival to first oral fluid intake, served as the primary outcome measurements for this study. Data was additionally collected regarding nausea and vomiting after returning to the ward, the incidence of postoperative complications, and the PACU observation time and hospitalization time.

## Materials and methods

### Sample

This prospective, randomized, controlled trial received approval from the Clinical trial and Biomedical Ethics Committee, West China Hospital, Sichuan University, Chengdu, China. This study was registered on the Chinese Clinical Trial Registry (ChiCTR-IOR-16008197) (http://www.chictr.org.cn/index.aspx. On April 2, 2016 the first patients was enrolled and on March 31, 2016 the trial was registered). From April 2016 – March 2018, a computer-generated list of random numbers served to mediate prospective randomization of a total of 2000 children corresponding to the American Society of Anesthesiology (ASA) I-III status who were in a post-anesthesia care unit (PACU) after general anesthesia. Exclusion criteria included: preoperative colds, respiratory infections, facial, oropharyngeal, thoracic, gastrointestinal, neuro-, or laryngeal surgery, altered mental state, or dysphagia.

On the basis of pilot study, a sample size of 628 allowed a 20% difference between the two groups, with an α of 0.05 (two-tailed) and a βof 0.20, power of 0.8. to account for attrition, a sample size of 785 was selected for each group.

### Study design

For all study participants, a complete medical history was taken, and patients and their parents were then informed of the study protocols (including the hydration regimens, follow-up plans, and thirst/satisfaction scoring systems). We additionally informed both groups of possible complications associated with the EOH treatment, such as nausea or vomiting. We then obtained written informed consent from the parents of all participating children. After surgical treatment was complete, patients were transferred to the PACU where they were monitored via pulse oximetry, electrocardiography, and noninvasive blood pressure monitoring. Patients were also provided with fluid intravenously. Thirst was rated verbally using a scale of 0–100, with the former representing no thirst and the latter representing the most significant possible thirst. Oropharyngeal discomfort similarly used a 0–100 scale, with 0 representing comfort and 100 representing the most worst discomfort ever suffered. One day postoperatively, patient satisfaction regarding their assigned treatment regimen was also assessed on a 0–100 scale from not satisfied to most satisfied.

A total of 2000 patients were randomized into the EOH and DOH groups (*n* = 1000/group). For the EOH group, medical professionals in the PACU monitored patient recovery until patients exhibited a normal mental status, degree V muscle recovery, stable vitals, and a normal swallowing reflex, at which time children were asked to consume water (< 5 ml/kg, 5% GS). The water was administered by a trained doctor or nurse by first aspirating the appropriate amount of water in an empty needle according to the child’s weight, tilting the child’s head to one side, and then slowly administering the water in the corner of the mouth. Patients in the DOH group were given oral water 4 h following anesthesia according to standard protocols. Immediately following recovery from anesthetization, patients were assessed for thirst and discomfort.

### Measures

Primary outcome and secondary outcome were recorded. Children over 5 years of age used self-reporting scales for thirst and hunger, with both scales extending from 0 to 100 (0: not hungry/thirsty; 100: very hungry/thirsty).

### Statistical analysis

SPSS v18.0 (SPSS Inc., Chicago, IL) was used for all analyses. Data are given as means ± SD. Continuous values were compared via Student’s t-tests and ANOVAs, while categorical variables were compared via chi-squared tests with Fisher’s test. *P* < 0.05 was the significance threshold.

## Results

**Fig. 1:**
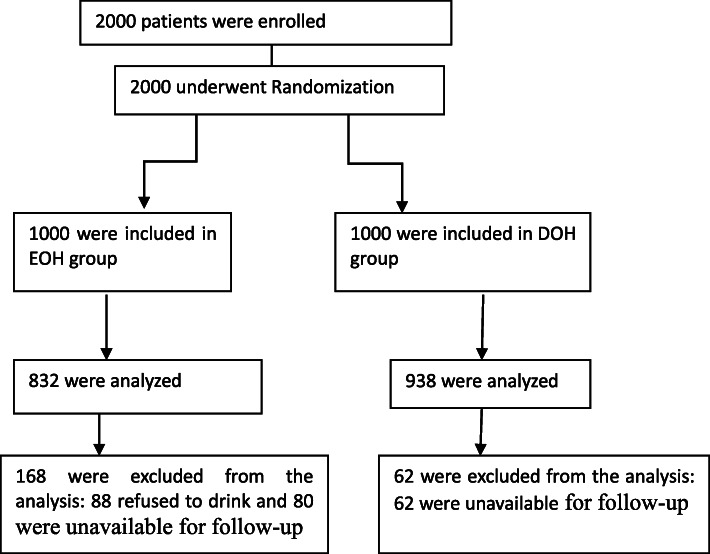
Randomization, treatment, and inclusion in Analysis. EOH group: early oral hydration group; DOH group: delayed oral hydration group

A total of 2000 children were enrolled in this study, with complete data being available for 1770 of these study participants. Of the remaining patients, 88 were excluded due to a failure to drink following anesthesia, while 142 were excluded due to their unavailability for follow-up (Fig. 1). Table 1 summarizes the demographics of the patients included in the final study dataset, with no significant differences in age, gender, weight, surgical operation, or ASA status between groups.

**Table 1:** Characteristics of the patient population and the surgical procedures performed

Characteristic and surgical procedure	Early oral hydration group (*n* = 832)	Delayed oral hydration group 4 h after operation group (*n* = 938)	P
Age (y)	5.59 ± 3.25	5.55 ± 3.21	0.79
Sex (%)			0.44
Male	483 (58.1)	562 (59.9)	
Female	349 (41.9)	376 (40.1)	
Weight (Kg)	21.51 ± 9.87	21.59 ± 10.20	0.87
Anesthesia time (min)	80.99 ± 51.45	78.53 ± 48.13	0.30
ASA (%)			0.88
I	179 (21.5)	194 (20.7)	
II	645 (77.5)	736 (78.5)	
III	8 (1.0)	8 (0.9)	
Type of peration			0.55
Polydactyly surgery	13 (1.6)	26 (2.8)	
Burn and plastic surgery	149 (17.9)	145 (15.5)	
orthopedic surgery	239 (28.7)	256 (27.3)	
Hemangioma surgery	22 (2.6)	29 (3.1)	
Ear surgery	43 (5.2)	49 (5.2)	
Ophthalmologic surgery	271 (32.6)	332 (35.4)	
Clubfeet surgery	6 (0.7)	11 (1.2)	
Ambulatory Stitches remove	12 (1.4)	11 (1.2)	
Hypospadias surgery	75 (9.0)	76 (8.1)	
Double J stent remove	2 (0.2)	3 (0.3)	0.75

*ASA* American Society of Anesthesiologists

Rates of nausea and vomiting were comparable between the DOH and EOH groups (*P* > 0.05) (Table 2). A similar finding was observed with respect to oropharyngeal discomfort scales (*p* < 0.0001). EOH group patients exhibited higher postoperative satisfaction scores (*P* < 0.001) (Table 3). With respect to the thirst and hunger scores of children over 5 years of age, compared with the DOH group, the scores of thirst and hunger in the EOH group at 10 and 20 min after general anesthesia were significantly lower (*P* < 0.05). After the patients were sent back to the ward, the thirst and hunger in the EOH group were significantly lower than those in the DOH group (*P* < 0.05) (Table 3).

**Table 2:** Drugs used during general anesthesia

Drugs	Early oral hydration group (*n* = 832)	Delayed oral hydration group (*n* = 938)	P
Propofol (%)	742 (89.2)	823 (87.7)	0.37
Fentanyl (%)	707 (85.0)	796 (84.9)	1.00
Midazolam (%)	717 (86.2)	790 (84.2)	0.26
Sevoflurane (%)	832 (100)	938 (100)	1.00
Sulfentanyl (%)	64 (7.7)	77 (8.2)	0.73
Remifentanil (%)	163 (19.6)	174 (18.6)	0.59
Cisatracurium Besilate (%)	534 (64.2)	600 (64.0)	0.96
Succinylcholine chloride (%)	5 (0.6)	6 (0.6)	1.00
Vecuronium bromide (%)	1 (0.1)	5 (0.5)	0.22
ketamine (%)	1 (0.1)	3(0.3)	0.63
Ropivacaine (%)	122 (14.7)	168 (17.9)	0.07
Ondansetron Hydrochloride (%)	656 (78.8)	748 (79.7)	0.68
Tropisetron Hydrochloride (%)	4 (0.5)	3 (0.3)	0.71
Neostigmine (%)	259 (31.1)	330 (35.2)	0.08

**Table 3:** Incidence of the postoperative nausea and vomiting in PACU and ward

	Early oral hydration group (*n* = 832)	Delayed oral hydration group (*n* = 938)	P
PACU nausea after 20 min (%)	1 (0.1)	5 (0.5)	0.22
PACU: vomiting after 20 min (%)	1 (0.1)	2 (0.2)	1.00
Ward: nausea after 4 h (%)	6 (0.7)	11 (1.2)	0.47
Ward: vomiting after 4 h (%)	5 (0.6)	7 (0.7)	0.78

## Discussion

The results of this randomized study indicate that EOH is safe in children immediately after general anesthesia associated with non-gastrointestinal surgery, significantly reducing thirst and discomfort while increasing satisfaction.

Postoperative hunger and thirst in children primarily occur due to prolonged preoperative fasting. In order to prevent reflux and aspiration during the operation, fasting for 2 h before anesthesia is necessary [9].

In this study, after recovery from general anesthesia, a small amount of water was given to children, and it did not increase the incidence of postoperative nausea or vomiting. This EOH treatment effectively lowered the thirst and hunger of children in the PACU, improving their comfort. Xiaoping Chen et al. found that the postoperative fasting time for patients undergoing non-abdominal surgery should be determined according to operation size, anesthesia method, and patient’s reactions following general anesthesia, with patients eating following recovery and elimination of nausea/vomiting symptoms [10]. Another study by Ding Chuanzhong et al. [11], included a control group given a gradually increasing fluid diet 6 h after operation, as well as a control group in which children without nausea and vomiting were evaluated and given warm water after they had recovered from general anesthesia. These children were first given 15–30 ml water, and, if they did not experience discomfort, some milk 15–30 min later, followed by glutinous rice porridge or noodles. In this study, the incidence of postoperative hyperpyrexia, thirst, hunger, crying, agitation, and family anxiety in the experimental group was significantly lower than that in the control group. We previously found it to be feasible for children to drink a small amount of GS after general anesthesia [12]. This work suggested that it was feasible and safe to drink a small amount of water after the recovery from general anesthesia once the cough and swallowing reflex had recovered.

Some researchers [13] have found that complete recovery of the swallowing reflex occurs within 24 min after anesthetization with propofol. Kerz et al. [14] concluded that it was safe to eat via the oral route at early time points after anesthetization when propofol is the only anesthetic used. Children have stomachs that empty rapidly, and preoperative fasting leads to hunger, crying, and significant swallowing of air, thereby leading to increased pressure in the stomach, abdominal distension, prolonged recovery of gastrointestinal peristalsis, and delayed recovery [15].

It is important that water intake in children be carefully monitored following general anesthesia. Children must be completely awake, with a recovered cough and swallowing reflex, as well as muscle strength that has recovered to grade V. When drinking water, children’s heads should be tilted to one side, and children should be monitored for cough, nausea, and hypoxemia. There were no instances of hypoxemia during this study. Doctors or nurses must carefully conduct this water administration, monitoring patient vital signs at all times. A small amount of water intake after recovery from general anesthesia can improve the comfort of children, improve their perioperative metabolic state, reduce thirst and hunger, reduce crying, and thereby help facilitate an earlier recovery.

### Limitations

Children did not drink or eat from 4 h after returning to the ward, based on standard protocols. There were no statistical differences in thirst or hunger scores from 2 to 4 h after returning to the ward. Further assessment of oral hydration should be continued after returning to the ward.

## Conclusions

Postoperative oral intake of fluids is very important for children following general anesthesia. This prospective randomized trial provided evidence that administering oral hydration at early post-anesthesia time points was safe in those children, improving satisfaction and decreasing thirst. Perioperative oral intake of fluids is very important for children following general anesthesia.

## Data Availability

The datasets used and/or analysed during the current study available from the corresponding author on reasonable request.

## Abbreviations

DOH: Late oral hydration
EOH: Early oral hydration
ASA: American Society of Anesthesiology
PACU: Postanesthesia care unit
ChiCTR: Chinese Clinical Trial Registry
